# Preformed Pt Nanoparticles Supported on Nanoshaped CeO_2_ for Total Propane Oxidation

**DOI:** 10.1021/acsanm.3c02688

**Published:** 2023-08-15

**Authors:** Shasha Ge, Yufen Chen, Xuan Tang, Yali Shen, Yang Lou, Li Wang, Yun Guo, Jordi Llorca

**Affiliations:** † Key Laboratory for Advanced and Research Institute of Industrial Catalysis, School of Chemistry & Molecular Engineering, East China University of Science and Technology, Shanghai 200237, P. R. China; ‡ Institute of Energy Technologies, Department of Chemical Engineering and Barcelona Research Center in Multiscale Science and Engineering, EEBE, Universitat Politècnica de Catalunya, Eduard Maristany 10-14, 08019 Barcelona, Spain; § Key Laboratory of Synthetic and Biological Colloids, Ministry of Education, School of Chemical and Material Engineering, Jiangnan University, Wuxi, Jiangsu 214122, P. R. China

**Keywords:** Pt nanoparticles, CeO_2_ facets, Pt−CeO_2_ interface, Pt chemical state, C_3_H_8_ oxidation

## Abstract

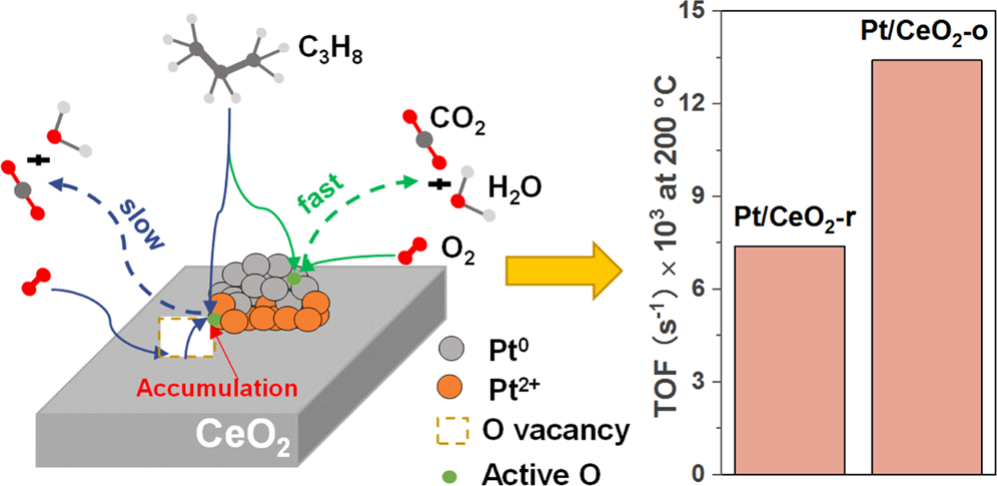

Pt-based catalysts have been widely used for the removal of short-chain volatile organic compounds (VOCs), such as propane. In this study, we synthesized Pt nanoparticles with a size of ca. 2.4 nm and loaded them on various fine-shaped CeO_2_ with different facets to investigate the effect of CeO_2_ morphology on the complete oxidation of propane. The Pt/CeO_2_-o catalyst with {111} facets exhibited superior catalytic activity compared to the Pt/CeO_2_-r catalyst with {110} and {100} facets. Specifically, the turnover frequency (TOF) value of Pt/CeO_2_-o was 1.8 times higher than that of Pt/CeO_2_-r. Moreover, Pt/CeO_2_-o showed outstanding long-term stability during 50 h. X-ray photoelectron spectroscopy (XPS) and diffuse reflectance infrared Fourier transform spectroscopy (DRIFTS) revealed that the excellent performance of Pt/CeO_2_-o is due to the prevalence of metallic Pt species, which promotes C–C bond cleavage and facilitates the rapid removal of surface formate species. In contrast, a stronger metal–support interaction in Pt/CeO_2_-r leads to easier oxidation of Pt species and the accumulation of intermediates, which is detrimental to the catalytic activity. Our work provides insight into the oxidation of propane on different nanoshaped Pt/CeO_2_ catalysts.

## 1 Introduction

The emissions of volatile organic compounds (VOCs) from mobile and stationary sources have raised concerns due to their hazardous effect on the environment and human health.^1−5^ Propane is widely regarded as one of the most representative VOCs due to its stable chemical nature and high bonding energy.^6−8^

A series of catalysts have been utilized for propane oxidation, including supported noble metal catalysts such as Pt,^9^ Pd,^10^ Ru,^11^ and transition-metal oxide catalysts such as Co,^12^ Mn,^13,14^ and Ni.^15^ Among these catalysts, Pt-based catalysts have been the preferred choice for the oxidation of propane because of their strong activation of C–H bonds and wide industrial applications.^16−20^ It has been reported that the chemical state of Pt plays an important role in the total oxidation of propane. Recently, Shan et al.^21^ revealed that the surface-oxygenated multimetallic alloy catalysts exhibited excellent activity for propane oxidation, which was attributed to the presence of partially positively charged Pt in combination with oxyphilic Ni–O and Co–O and then further facilitated breaking of C–C bonds of C_3_H_8_ and the elimination of reaction intermediates on the catalyst surface. Huang et al.^22^ reported a Pt/Nb_2_O_5_ catalyst for the complete combustion of propane and found that a higher concentration of metallic Pt species facilitated the heterolytic splitting of C–H bonds in propane through the formation of chemisorbed oxygen species (O*) on the metallic Pt surface at stable Pt^δ+^–(O*)^δ−^ dipolar sites.

CeO_2_-based catalysts are considered as highly promising candidates for eliminating VOC pollutants due to their exceptional oxygen storage and unique redox properties.^23−27^ The modulation of CeO_2_ morphology, especially by exposure of specific nanocrystalline facets, is a prevalent approach to optimize surface structures, thereby improving its catalytic activity. Besides, many studies have shown that the crystal orientation of CeO_2_ has strong effects on the chemical state of noble metals on CeO_2_. For example, Pt supported on different nanoshaped CeO_2_ terminated predominantly by {100}, {110}, or {111} surfaces for the production of 1,2-pentanediol from furfuryl alcohol has been reported, where Pt chemical states are controlled by the facets of CeO_2_ and metallic Pt with a small particle size plays a significant role in the prominent catalytic activity.^28^ The same phenomenon has been reported on Au/CeO_2_ for COPrOx and CO oxidation and the remarkably enhanced activity is attributed to oxidized Au controlled by CeO_2_ morphologies.^29^ Similarly, Pd and Ru-supported CeO_2_ catalysts showed ceria-shape-dependent activities for propane and CO oxidation.^30,31^ Very recently, Pt supported on different CeO_2_ facets prepared by incipient wetness impregnation (IWI) has been studied for propane oxidation.^32^ In this research, the nano-lamellar CeO_2_ with the {110} facet showed higher activity after aging and reduction pretreatment in comparison to nano-cube CeO_2_ with the {100} facet because of the presence of particular opportune Pt size/Pt charge and proper amount of oxygen vacancies. It is worth noting that the impregnation method can lead to the coexistence of many types of Pt structures (such as single atoms, clusters and nanoparticles (NPs)) with a large Pt size distribution, and the process of pretreatment with H_2_ also could affect the chemical state of Pt. In particular, the chemical states of the metals, which are affected by the particle size, have been proven to play a crucial role in catalytic performance.^33−36^ In order to clarify the influence of different facets of CeO_2_ on the Pt state and propane activity, we used the ethylene glycol reduction method to control the particle size of supported Pt nanoparticles. Under the condition of the same size of Pt nanoparticles, the effects of interaction between Pt and different facets of CeO_2_ on the Pt state and catalytic activity were investigated.

Herein, we have prepared Pt nanoparticles (NPs) with a mean particle size of ca. 2.4 nm and subsequently supported them on nanoshaped CeO_2_ (rods and octahedral) to carefully investigate their structure–property relationships for propane oxidation. The use of preformed Pt NPs ensured that all samples had the same Pt particle size and a similar Pt–support interface perimeter, which allowed us to elucidate the precise role of the shape of the cerium support on the complete oxidation of propane. The higher activity for propane oxidation was achieved on Pt/CeO_2_-o with {111} facets compared to Pt/CeO_2_-r exposing the {110} and {100} facets. It was found that the chemical state of Pt is governed by the facets of CeO_2_ through different intensities of the Pt–CeO_2_ interaction. This work highlights the important role of the terminal surface of cerium dioxide in the Pt/CeO_2_ system in regulating the chemical state of Pt and its effect on the combustion of propane.

## 2 Experimental Section

### 2.1 Catalyst Preparation

**Scheme 1:**
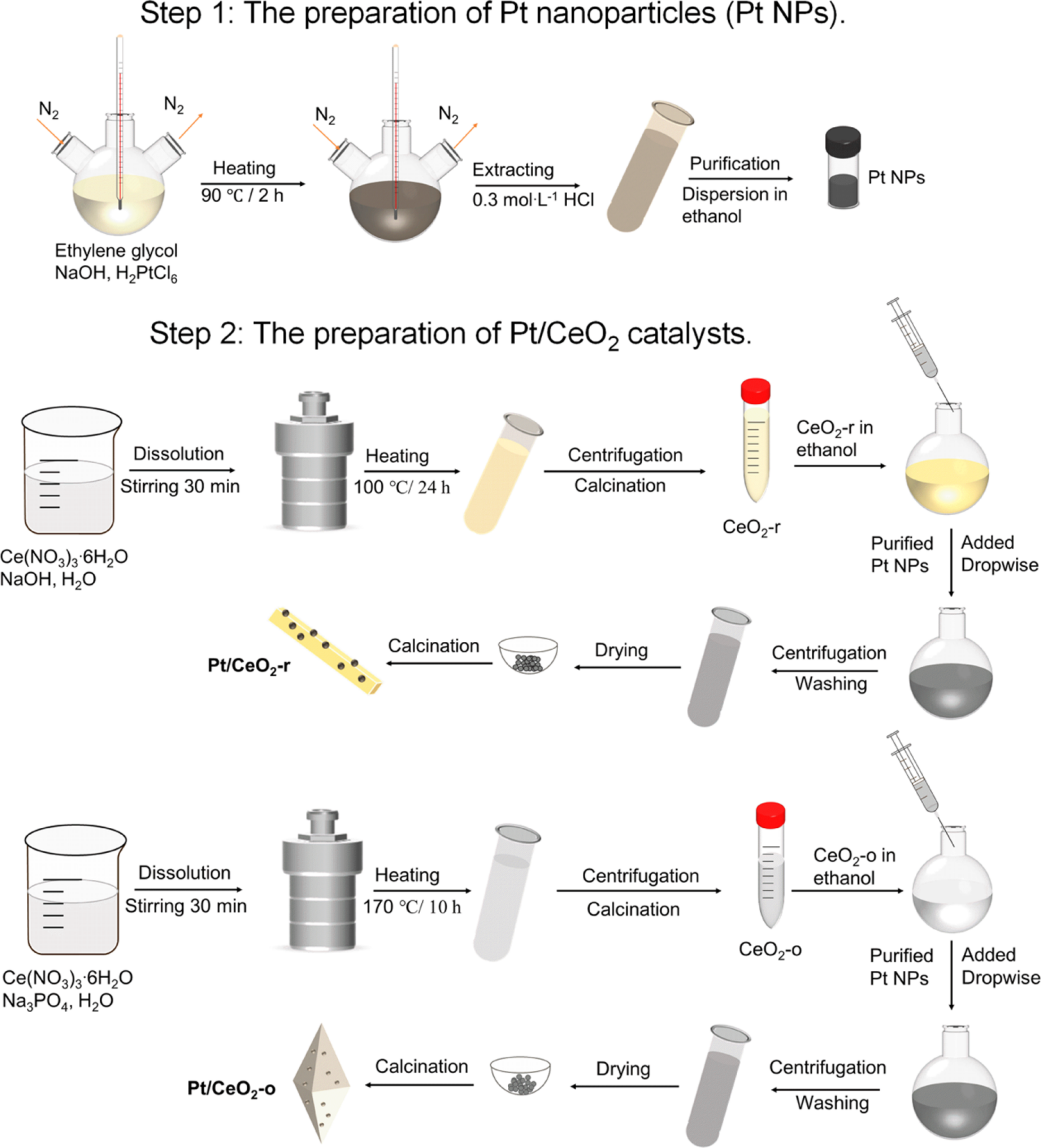
Illustration of Catalyst Preparation

Ceria nanorods (denoted as CeO_2_-r) and nano-octahedrons (denoted as CeO_2_-o) were synthesized by hydrothermal methods.^31,37^ The synthesis of ceria rods (CeO_2_-r) and ceria octahedrons (CeO_2_-o) is mentioned in previous papers published by our group.^10^ Briefly, 10 mL of aqueous 0.4 M Ce(NO)_3_·6H_2_O was mixed with 70 mL of 6.8 M NaOH solution and continually stirred for 30 min. Then, the suspension was transferred to the hydrothermal reactor and heated at 100 °C for 24 h to obtain CeO_2_-r. For the ceria octahedrons, 10 mL of 0.19 M Ce(NO)_3_·6H_2_O and 70 mL of 6.6 × 10^–4^ M Na_3_PO_4_ aqueous solution were mixed and vigorously stirred for 30 min. The solution was hydrothermally treated at 170 °C for 10 h. After cooling, the obtained solid was separated and washed several times with deionized water and ethanol, then dried at 80 °C for 8 h and finally calcined at 400 °C for 4 h. Pt nanoparticles were prepared by the ethylene glycol reduction method.^38,39^ The adsorption method was used to deposit Pt on CeO_2_, labeled Pt/CeO_2_-r and Pt/CeO_2_-o. Pt/CeO_2_-r-IWI was prepared by incipient wetness impregnation (IWI). Further details can be found in the Supporting Information. Their synthetic routes are shown in Scheme 1.

### 2.2 Catalyst Characterization

Powder X-ray diffraction (XRD) patterns were obtained on a Rigaku D/Max-rC diffractometer with Cu Ka radiation (λ = 1.5418 Å) from 10 to 80° at 40 kV and 40 mA. The mean crystallite size of samples was calculated by the Scherrer equation. N_2_ adsorption–desorption isotherms were obtained on a Micromeritics ASAP 2020M instrument. The surface area of the samples was calculated by the Brunauer–Emmett–Teller (BET) method. PerkinElmer Optima 2100 DV inductively coupled plasma-atomic emission spectroscopy (ICP-AES) was employed to detect the content of Pt. Transmission electron microscopy (TEM) images were obtained on a JEOL model 2100F electron microscope, which operates at 200 kV. X-ray photoelectron spectroscopy (XPS) was performed on a VG ESCALAB MK II system equipped with a hemispherical electron energy analyzer. The C 1s signal at 284.8 eV was used to calibrate the binding energy and Casa XPS software was used to analyze the results.

H_2_ temperature-programmed reduction (H_2_-TPR) was performed on a Pengxiang PX200 instrument (Tianjin, China) equipped with a thermal conductivity detector (TCD). 50 mg of the catalyst was placed into a U-quartz reactor and heated from 25 to 800 °C under a stream of 50 mL·min^–1^ 5% H_2_/N_2_ at a rate of 10 °C·min^–1^. H_2_ consumption was quantified using the H_2_ consumption of pure CuO as a calibration standard. The Pt dispersion was determined by the CO-pulse absorption method on a Micromeritics Autochem II 2920 chemisorption analyzer equipped with a mass spectrometer (MS). First, the catalyst was pretreated with 10% H_2_/He for 1 h at 300 °C and then cooled to 25 °C in a He stream. After that, a certain amount of 1% CO/He (0.5173 mL) was injected into the reactor every 4 min until no CO consumption was observed. An atomic ratio of CO to exposed Pt was assumed to be 1:1 to calculate the dispersion of Pt. Temperature-programed desorption of O_2_ (O_2_-TPD) was performed on the above chemisorption equipment and the signal of O_2_ (*m*/*z* = 32) was followed by MS. Typically, the catalyst (50 mg) was pretreated in 3% O_2_/He (40 mL·min^–1^) at 400 °C for 1 h, then cooled to room temperature under this mixture gas, and purged with He (40 mL·min^–1^) for 1 h. Finally, the sample was heated to 600 °C at a rate of 10 °C·min^–1^. The temperature-programmed oxidation (O_2_-TPO) was performed on the same apparatus as O_2_-TPD and the signal of CO_2_ (*m*/*z* = 44) was followed by MS. After a long-term stability test, the catalyst (50 mg) was placed in a U-tube. After the baseline of MS profile remained stable, the catalyst was heated from room temperature to 800 °C under 3% O_2_/He (50 mL·min^–1^) conditions with a temperature ramp of 10 °C·min^–1^.

To measure the amount of adsorbed surface oxygen, 50 mg of the sample was reduced at 400 °C for 40 min in 10% H_2_/He (50 mL·min^–1^) on the chemisorbed equipment described above, then cooled down to room temperature and purged with He for 30 min. A certain amount of 3% O_2_/He (0.5173 mL) was pulsed every 4 min until the equilibrium was achieved. The consumption of oxygen by catalyst was defined as OSC_catalyst_. OSC_Pt_ represents the consumption of oxygen by Pt and was calculated by the following equation.
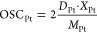
1where *X*_Pt_ is the content of Pt tested by ICP-AES; *D*_Pt_ is the dispersion of Pt; *M*_Pt_ is the atomic weight of Pt (195.1 g·mol^–1^); and the stoichiometric factor between the metal atom and oxygen atom is set at 1:2. The value of surface oxygen vacancy concentration was defined as OSC_surface_ and calculated by the following equation.
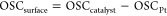
2*Insitu* diffuse reflectance infrared Fourier transform spectroscopy (DRIFTS) was measured on a Nicolet Nexus 6700 spectrometer with 64 scans at an effective resolution of 4 cm^–1^. Briefly, the sample cell was heated to 220 °C under Ar (50 mL·min^–1^) and purged 40 min before background collection, and then the mixed gas 0.2% C_3_H_8_/Ar was introduced at a flow rate of 50 mL·min^–1^. At the stage of C_3_H_8_ oxidation, a mixture of 0.2% C_3_H_8_/2% O_2_/Ar (50 mL·min^–1^) was introduced into the chamber. For the desorption, the reaction gas was switched to Ar. All spectra were collected after 30 min. The CO-DRIFTS was conducted at room temperature. Before measurement, the sample was pretreated at 220 °C under Ar and then cooled to room temperature. Then, the CO adsorption spectra were recorded under CO/Ar (50 mL·min^–1^) until fully saturated. Finally, the CO/Ar mixture was replaced with Ar to remove the gas-phase adsorption of CO.

### 2.3 Evaluation of Catalytic Activity

100 mg of the catalyst with 200 mg of inert quartz sand (40–60 mesh) was physically mixed and placed into a fixed-bed quartz reactor for the total oxidation of C_3_H_8_. The reaction gas consisted of 0.2% C_3_H_8_, 2% O_2_, and Ar (50 mL·min^–1^) passing through the catalytic bed at a weight hourly space velocity (WHSV) of 30 000 mL·h^–1^·g^–1^. The temperature was programmed from 100 to 400 °C at 2 °C·min^–1^ and the concentration of C_3_H_8_ was measured by an online gas chromatograph (GC-9790) equipped with a flame ionization detector (FID). The stability of the catalysts was evaluated at the temperature up to 90% conversion of propane (*T*_90_). The C_3_H_8_ conversion (*X*_C_3_H_8__) was calculated by the following formula.
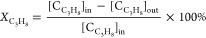
3where [C_C_3_H_8__]_in_ and [C_C_3_H_8__]_out_ are the C_3_H_8_ concentrations in the inlet and outlet gases, respectively.

### 2.4 Reaction Kinetics Measurement

The kinetic data for the propane oxidation was obtained by controlling the propane conversion below 15%. The specific reaction rate of C_3_H_8_ (*r*_C_3_H_8__) was calculated using the following equation.
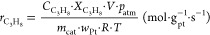
4where *C*_C_3_H_8__ is the concentration of C_3_H_8_ in the feed gas, *X*_C_3_H_8__ is the conversion of C_3_H_8_ (%), *V* is the total flow, *m*_cat_ is the mass of the catalyst used, w_Pt_ represents the Pt content measured by ICP-AES.

The turnover frequency (TOF) was calculated by the following equation.
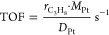
5where *r*_C_3_H_8__ is the reaction rate of propane oxidation at *t* = 10 min, *M*_Pt_ is the atomic weight of Pt (195.1 g·mol^–1^), and *D*_Pt_ is the dispersion of Pt calculated by the CO-pulse.

## 3 Results and Discussion

**Figure 1:**
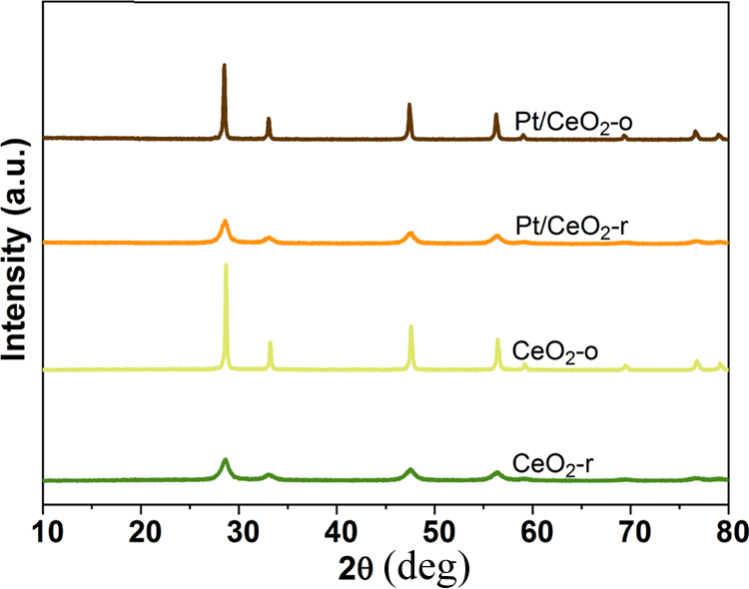
XRD patterns of pure CeO_2_ and Pt/CeO_2_ catalysts.

The crystalline structures of shape-controlled CeO_2_ and Pt/CeO_2_ were confirmed by XRD. As shown in Figure 1, in all samples, only the structure of the face-centered cubic fluorite-type (JCPDS File Card No. 34-0394) phase ascribed to ceria was observed. The diffraction peaks of CeO_2_-o are higher and sharper than those of CeO_2_-r, indicating big grain sizes and higher crystallinity.^32^ No diffraction peaks associated with Pt (39.8°) species were detected in the XRD patterns of Pt/CeO_2_, due to the low metal loading or small Pt NP size.^11^ In addition, the particle sizes of CeO_2_ measured from TEM images are (8 ± 2) × (120 ± 38) and 117 ± 19 nm for CeO_2_-r and CeO_2_-o, respectively (Table 1). The specific surface area of CeO_2_-r is 99 m^2^·g^–1^, which is about 10 times higher than that of CeO_2_-o (10 m^2^·g^–1^), and they decrease slightly after loading Pt. The actual Pt loading in Pt/CeO_2_ was measured by ICP-AES. As shown in Table 1, the loading of Pt on the surface of ceria nanorods is 1 wt %, in agreement with the theoretical loading. However, for Pt/CeO_2_-o, the Pt content is 0.38 wt %, which is significantly lower than the theoretical load due to the loss of Pt during the washing process.

**Table 1:** Pt Loading, BET Specific Surface Area (*S*_BET_), CeO_2_ Crystallite, Particle Size, and Pt Size

samples	Pt loading (wt %)^a^	*S*_BET_ (m^2^·g^–1^)	CeO_2_ crystallite size (nm)^b^ XRD	CeO_2_ particle size (nm) TEM	Pt size (nm) TEM
CeO_2_-r		99	9.3	(8 ± 2) × (120 ± 38)	
CeO_2_-o		10	38.2	117 ± 19	
Pt/CeO_2_-r	1.00	85	8.9		2.4 ± 0.1
Pt/CeO_2_-o	0.38	8	36.5		2.3 ± 0.1

a Measured by ICP-AES.

b Calculated by Scherrer equation.

**Figure 2:**
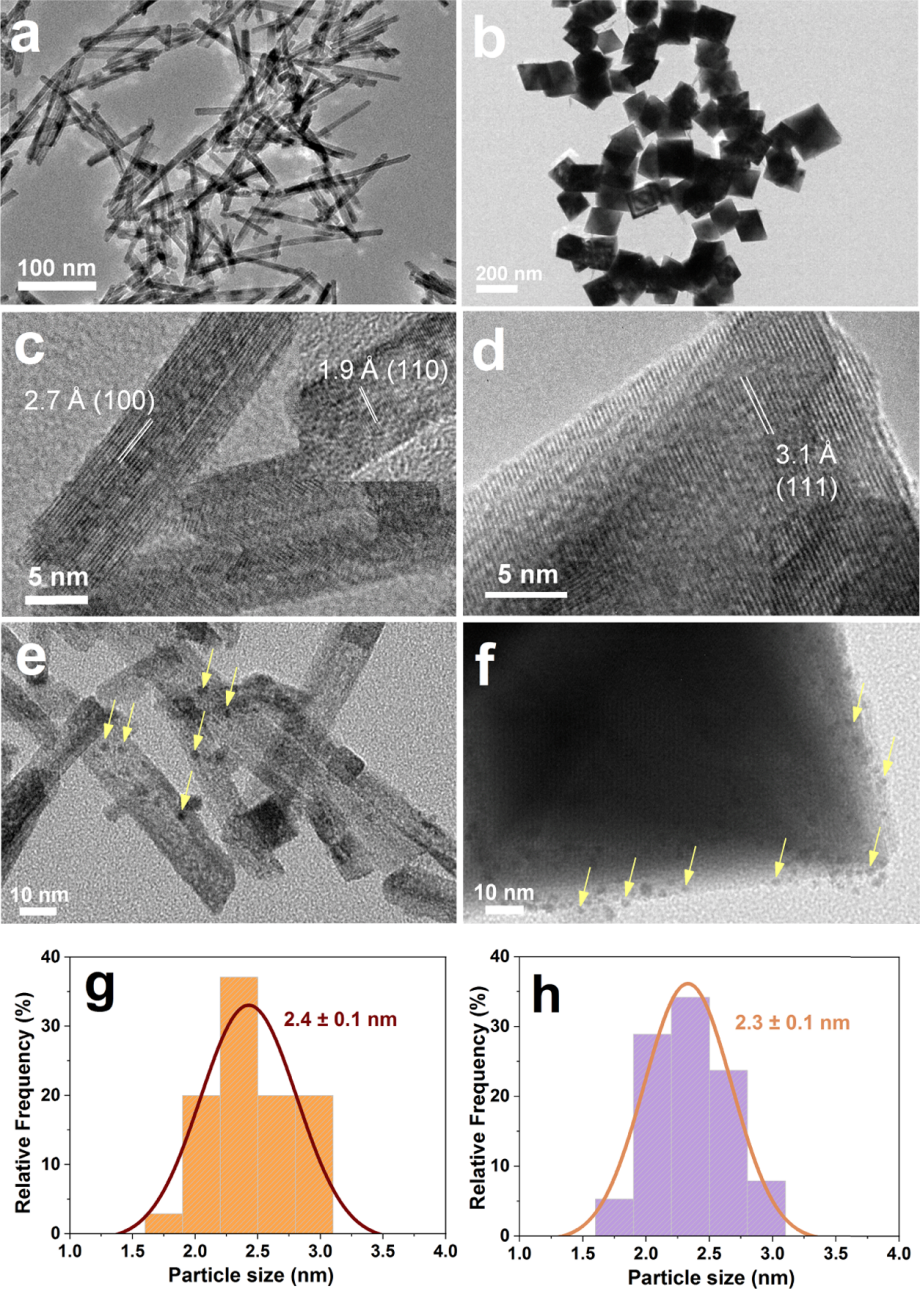
TEM and HRTEM images of CeO_2_-r (a, c), CeO_2_-o (b, d), Pt/CeO_2_-r (e), and Pt/CeO_2_-o (f); the particle size distribution histograms of Pt/CeO_2_-r (g) and Pt/CeO_2_-o (h).

TEM and high-resolution TEM (HRTEM) were employed to analyze the morphologies of various Pt/CeO_2_ samples and the size distribution of Pt NPs, as shown in Figure 2. All CeO_2_ shows well-defined shapes confirming the formation of CeO_2_-r and CeO_2_-o (Figure 2a,b). As revealed by HRTEM, CeO_2_-r exhibits well-defined {110} and {100} terminal facets with a lattice spacing of 1.9 and 2.7 Å, respectively (Figure 2c), as previously reported.^10^ For octahedral ceria, only the {111} facets with an interplanar spacing of 3.1 Å was detected (Figure 2d), which means that the nano-octahedron is mainly enclosed by the {111} facets.^40^ From Figure S1, it can be seen that Pt NPs in colloidal solution have an average size of 2.4 ± 0.1 nm. After loading, Pt NPs (marked with yellow arrows) are highly dispersed on the surface of CeO_2_, and no large Pt particles were observed, which is consistent with the XRD results. The average particle sizes of Pt NPs on CeO_2_-r and CeO_2_-o were measured to be 2.4 ± 0.1 and 2.3 ± 0.1 nm, respectively (Figure 2g,h), confirming the similar size of Pt on the different morphologies of CeO_2_.

**Figure 3:**
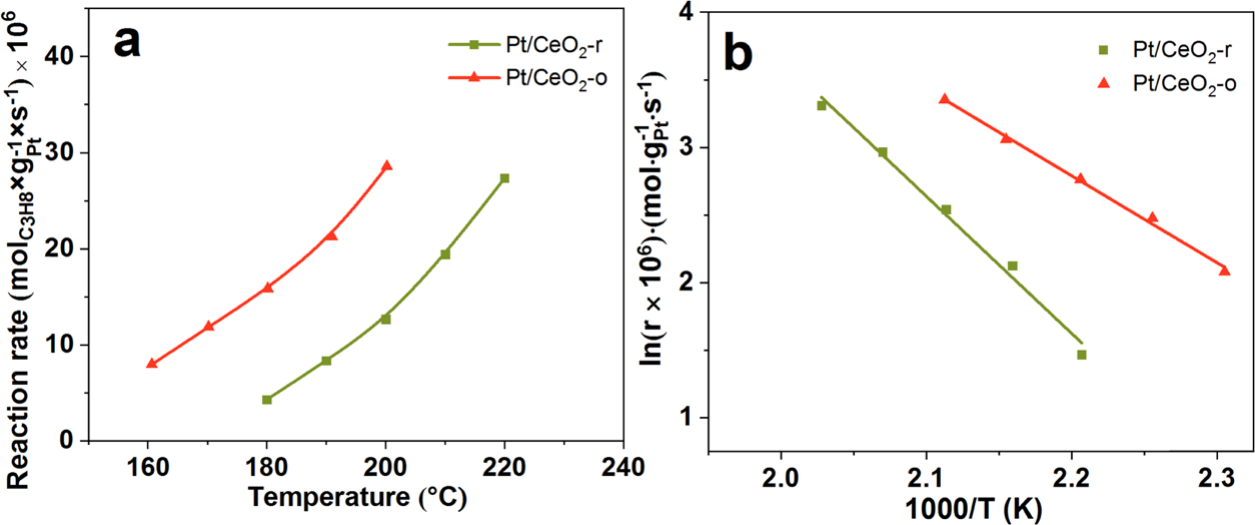
Reaction rate (a) and Arrhenius plots (b) of the Pt/CeO_2_ catalysts.

The catalytic performance of Pt/CeO_2_ samples was examined for total propane oxidation as shown in Figures 3a and S2, and the specific reaction rate (*r*) was calculated as the amount of propane consumed per gram of Pt per second. The reduced CeO_2_-r support was significantly more active toward propane oxidation than the reduced CeO_2_-o. As expected, the presence of platinum strongly promoted the propane oxidation. Remarkably, for the same Pt content (0.38%), Pt/CeO_2_-o showed much higher propane oxidation activity than Pt/CeO_2_-r (Figure S2). As listed in Table 2, the *r* of Pt/CeO_2_-o is 28.56 × 10^–6^ mol·g_Pt_^–1^·s^–1^ at 200 °C, which is about twice as much as that of Pt/CeO_2_-r (12.68 × 10^–6^ mol·g_Pt_^–1^·s^–1^). Besides, the calculated TOF value of Pt/CeO_2_-o (13.40 × 10^–3^ s^–1^) is approximately 1.8 times higher than that of Pt/CeO_2_-r (7.38 × 10^–3^ s^–1^), suggesting that Pt species on the CeO_2_-o is much more active with respect to that on CeO_2_-r. This result indicates that the performance of Pt/CeO_2_ catalysts for propane oxidation is closely related to the characteristics of the different morphologies of CeO_2_ and Pt/CeO_2_-o has a superior activity compared with Pt/CeO_2_-r. Based on this data, the Arrhenius plots over Pt/CeO_2_ catalysts were obtained and the apparent activation energy (*E*_a_) was determined (Figure 3b). The *E*_a_ of Pt/CeO_2_-o (53.5 ± 1.9 kJ·mol^–1^) is much lower than that of Pt/CeO_2_-r (84.4 ± 4.9 kJ·mol^–1^), which indicates that the reaction pathways for propane oxidation on these two catalysts are different.

**Table 2:** Pt Dispersion, Reaction Rate (*r*), TOF and *E*_a_ Values of Pt/CeO_2_-r and Pt/CeO_2_-o Catalysts

samples	Pt dispersion (%)^a^	*r* × 10^6^ (mol·g_Pt_^–1^·s^–1^) at 200 °C	TOF (s^–1^)^b^ × 10^3^	*E*_a_ (kJ·mol^–1^)
Pt/CeO_2_-r	33.5	12.68	7.38	84.4 ± 4.9
Pt/CeO_2-_o	41.5	28.56	13.40	53.5 ± 1.9

a Obtained from CO chemisorption experiments.

b Calculated at 200 °C.

Meanwhile, to clarify the promoting effect of Pt NPs in propane oxidation, Pt/CeO_2_-r-IWI with 1 wt % platinum content was prepared by the impregnation method (IWI). Before testing, this IWI catalyst was reduced at 300 °C for 90 min under 10% H_2_/N_2_ conditions to activate the catalyst. As shown in Figure S3a, the activity of Pt/CeO_2_-r is superior to that of the Pt/CeO_2_-r-IWI sample. Considering the presence of moisture conditions in the practical operation process, 3% H_2_O was introduced to investigate the effect of water on the activity of the catalysts. As shown in Figure S3, the *T*_90_ of Pt/CeO_2_-r-IWI increased from 327 to 393 °C (66 °C), whereas for Pt/CeO_2_-r and Pt/CeO_2_-o, the *T*_90_ increased only by 22 and 29 °C, respectively. This indicates that Pt/CeO_2_-r not only has higher activity but also weakens the effect of water compared to the IWI sample. This observation is significant taking into account that the Pt dispersion in Pt/CeO_2_-r-IWI is higher than that of Pt/CeO_2_-r (62.0 vs 33.5%).

**Figure 4:**
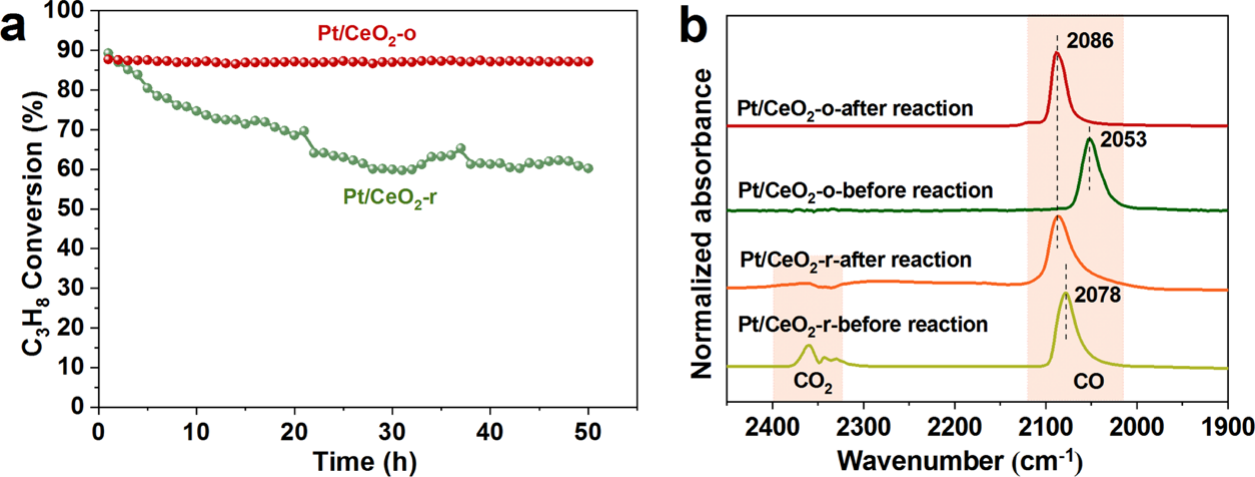
Long-term stability for propane oxidation over Pt/CeO_2_ (a) and DRIFTS of CO adsorption for the fresh and used Pt/CeO_2_ sample (b).

To evaluate the durability of Pt/CeO_2_ for propane oxidation, long-term stability tests were carried out on Pt/CeO_2_-r and Pt/CeO_2_-o at *T*_90_ within 50 h. As shown in Figure 4a, the propane conversion over the Pt/CeO_2_-o sample is perfectly maintained within 50 h, while that over Pt/CeO_2_-r decreased from 90 to 49% with the extension of time, indicating that the Pt/CeO_2_-o catalyst has excellent long-term stability for total propane oxidation. Additionally, to explore the cause of deactivation, CO-DRIFTS was used to study the change of Pt species before and after the long-term stability test on Pt/CeO_2_-r and Pt/CeO_2_-o catalysts. CO-DRIFTS was first performed on the fresh samples (before the reaction), followed by the introduction of the reaction gas (C_3_H_8_/O_2_/Ar) into the infrared chamber for 10 h (after the reaction). As shown in Figure 4b, for the fresh sample, the adsorption of CO at 2078 and 2053 cm^–1^ can be ascribed to CO absorbed on under-coordinated metallic Pt sites.^41^ For the used sample, the peak at 2086 cm^–1^ can be attributed to the adsorption of CO on well-coordinated metallic Pt sites, which indicates that the surface structure of Pt changed after the propane oxidation stability tests.^42^ The same position of CO adsorption peaks for the used samples suggests that changes in the structure of Pt are not responsible for the deactivation of Pt/CeO_2_-r. The cause of deactivation of the Pt/CeO_2_-r will be explored in the next section. Combining the activity and the stability tests, we can conclude that the facets of CeO_2_ not only affect the activity of the catalyst but also has an impact on the stability of the catalyst for propane oxidation.

**Figure 5:**
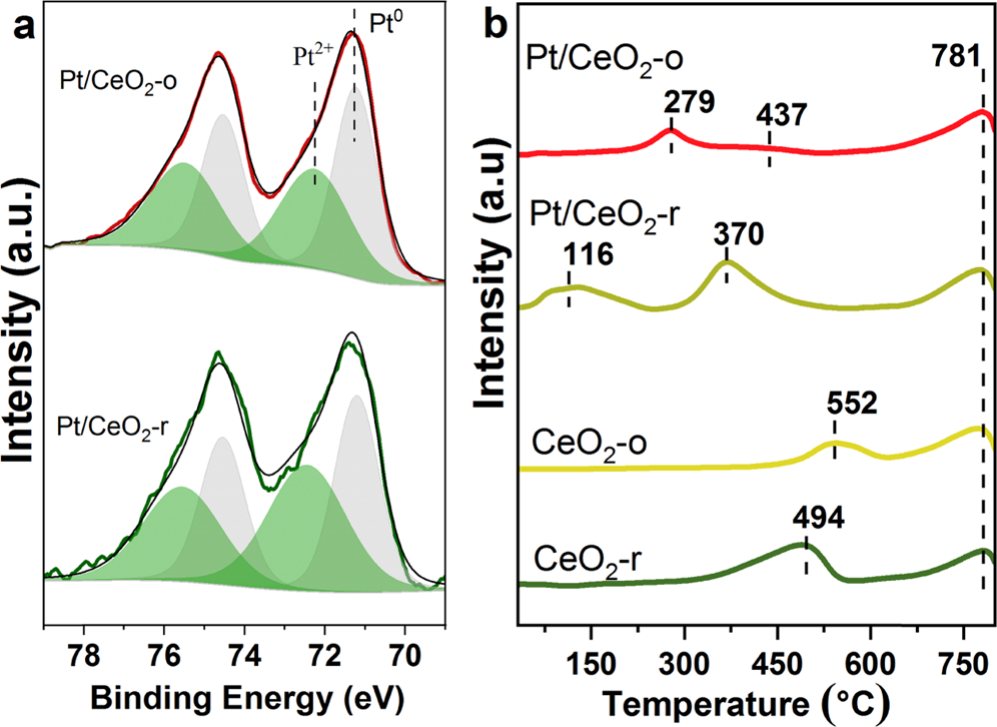
XPS spectra of Pt 4f (a) and H_2_-TPR (b) of CeO_2_ and Pt/CeO_2_ catalysts.

It has been suggested that the performance of catalysts is significantly influenced by the chemical state of noble metals.^9,43^ In order to clarify the relationship of catalytic activity with the chemical state of Pt, the XPS spectra of Pt 4f were performed and shown in Figure 5a. The surface atomic concentration of Pt on the Pt/CeO_2_-o (1.0%) is higher than that of Pt/CeO_2_-r (0.3%). After careful consideration, the lower surface atomic ratio of Pt on Pt/CeO_2_-r compared to Pt/CeO_2_-o should be related to the larger surface area of rod-shaped ceria. As shown in Figure 5a, the fitted peaks at 71.2 and 72.3 eV for Pt 4f_7/2_ can be assigned to Pt^0^ and Pt^2+^, respectively.^44,45^Table 3 summarizes the ratio of Pt^0^/(Pt^0^ + Pt^2+^) estimated from the Pt 4f signal. The ratio of Pt^0^ on Pt/CeO_2_-r (46.6%) is significantly lower than that of the Pt/CeO_2_-o (59.2%) catalyst. This result reveals that the metallic state Pt is more preserved on the CeO_2_-o with {111}. It has been reported that the metallic state platinum facilitated the cleavage of C–H and C–C bonds and enhanced the activity of propane oxidation.^6,9^

**Table 3:** H_2_ Uptake in H_2_-TPR, OSC and XPS Results

samples	peak position (25–600 °C)	consumption of H_2_ (μmol·g^–1^)	OSC_catalyst_ (μmol·[O]·g^–1^)	OSC_Pt_ (μmol·[O]·g^–1^)	OSC_surface_ (μmol·[O]·g^–1^)	Pt^0^/Pt (%)	Ce^3+^/Ce (%)
CeO_2_-r	494	429					
CeO_2_-o	552	137					
Pt/CeO_2_-r	116, 370	219, 442	390.2	34.3	355.9	46.6	21.0
Pt/CeO_2_-o	279, 437	120, 10	40.5	16.2	24.3	59.2	13.8

The strength of the interaction between noble metal and support has been reported to be correlated with the metal’s chemical state.^46^ Therefore, to investigate the strength of the interaction between Pt and CeO_2_ with different morphologies, H_2_-TPR was carried out and the results are shown in Figure 5b and Table 3. The reduction of surface lattice oxygen occurs at 494 and 552 °C for CeO_2_-r and CeO_2_-o, respectively,^47^ and CeO_2_-r shows a higher H_2_ consumption (429 μmol·g^–1^) compared to CeO_2_-o (137 μmol·g^–1^) (Table 3). These results show that the reduction of surface lattice oxygen is related to the shape of ceria, which is associated with the oxygen vacancy formation energies following the order {110} < {100} < {111}.^23,48^ The peak at 781 °C can be assigned to the reduction of lattice oxygen in bulk ceria.

For Pt/CeO_2_-o, there are two reduction peaks, one peak at 279 °C and another very weak peak at 437 °C, and the total consumption amount of H_2_ is nearly consistent with that of reduction of surface lattice oxygen species on CeO_2_-o. According to previous research, this peak can be regarded as a reduction of surface oxygen from CeO_2_,^28,49^ the presence of Pt NPs promote this reduction process due to the H_2_ spillover. For the reduction of Pt/CeO_2_-r, a peak was observed on Pt/CeO_2_-r at 116 °C with H_2_ consumption of 219 μmol·g^–1^, which is higher than the theoretical value for the reduction of Pt^4+^ to Pt^0^ (102 μmol·g^–1^). Therefore, this peak can be attributed to the co-reduction of the PtO*_x_* and CeO_2_ at the Pt–CeO_2_ interface due to the strong interaction effect (Table 3).^28^ The peak at 370 °C is usually attributed to the reduction of surface oxygen species adjacent to Pt species, which is lower than the reduction temperature of pure CeO_2_ (494 °C) due to the H_2_ spillover. The results of H_2_-TPR show that the strength of the Pt–CeO_2_ interaction and the reducibility of Pt/CeO_2_ catalysts depends on the morphology of CeO_2_ and the stronger interaction between Pt and CeO_2_ is confirmed on Pt/CeO_2_-r. The XPS and H_2_-TPR results show that the different crystal facets of CeO_2_ can regulate the electronic state of Pt through the Pt–CeO_2_ interaction and that a weak interaction is more likely to keep Pt in a metallic state and thus more active for propane oxidation.

**Figure 6:**
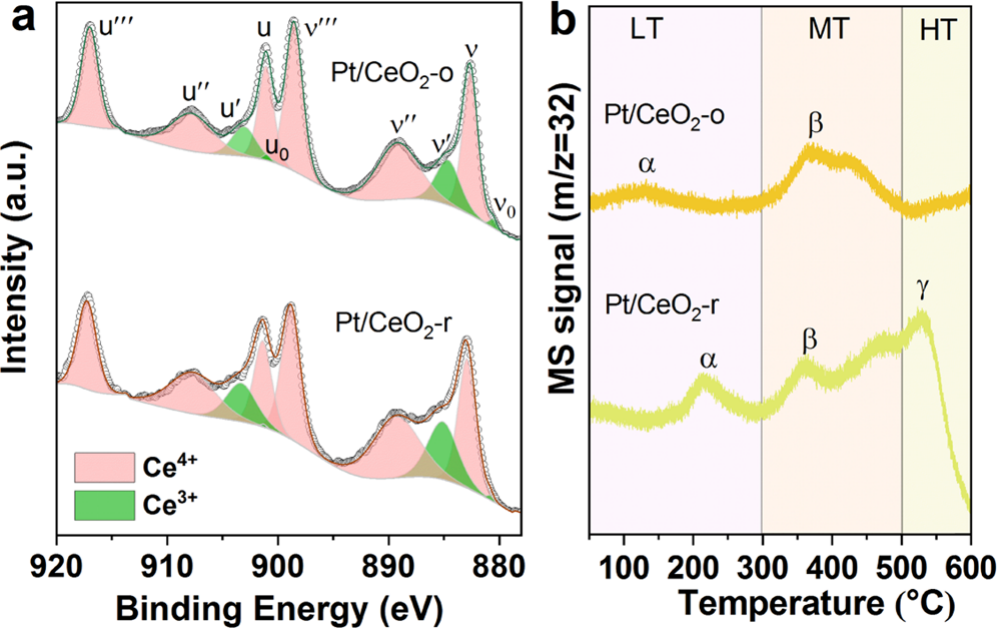
XPS spectra of Ce 3d (a) and O_2_-TPD (b) profiles for Pt/CeO_2_ catalysts.

The oxygen vacancies can not only stabilize the noble metal but also provide the activation oxygen for propane oxidation. It has been shown that more oxygen vacancies on Ru/CeO_2_ lead to higher adsorption and activation of oxygen to promote propane oxidation.^31^ Therefore, the Ce 3d XPS and Raman spectra were employed to confirm the concentration of the oxygen vacancies. The Ce 3d XPS are shown in Figure 6a, which are resolved into 10 peaks. According to the previous literature, the peaks assigned to Ce^4+^ are labeled as u‴ (916.9 eV), v‴ (898.4 eV), u (901.1 eV), v (882.6 eV), u″ (907.5 eV), and v″ (888.9 eV), while the peaks u′ (902.6 eV), u_0_ (899.4 eV), v′ (884.4 eV), and v_0_ (881.1 eV) are assigned to Ce^3+^. Generally, the concentration of Ce^3+^ is correlated to the surface oxygen vacancies.^50−52^ As listed in Table 3, the surface ratio of Ce^3+^ in Pt/CeO_2_-r (21.0%) is higher than in Pt/CeO_2_-o (13.8%), indicating that more intrinsic defect sites and oxygen vacancies on the surface of Pt/CeO_2_-r. Figure S4 shows normalized Raman spectra of CeO_2_ and Pt/CeO_2_. The most intense peak at 461 cm^–1^ corresponds to the first-order F_2g_ symmetry of CeO_2_.^51^ The weak peak at 598 cm^–1^ is attributed to the defect-induced vibration mode (D).^15,31^ Compared with Pt/CeO_2_-o, Pt/CeO_2_-r has a more prominent peak at 598 cm^–1^, indicating a higher oxygen vacancy in Pt/CeO_2_-r, which is consistent with the XPS data.

To investigate the mobility of oxygen species, O_2_-TPD was performed in Figure 6b. For Pt/CeO_2_-r, three peaks are observed. The desorption peak below 300 °C (α) can be assigned to the desorption of oxygen on Pt NPs (O@Pt). The lower α temperature was observed on Pt/CeO_2_-o compared to Pt/CeO_2_-r, which corresponds to the result of H_2_-TPR, where the oxygen on Pt was reduced at room temperature. In addition, the α area of Pt/CeO_2_-o is smaller than that of Pt/CeO_2_-r because of the lower loading of Pt and the tendency of Pt to exist in the metallic state on CeO_2_-o, while higher Pt loading and more Pt in the partially oxidized state are present on CeO_2_-r. The peak between 300 and 400 °C (β) can be attributed to the desorption of the surface lattice oxygen on the interface of Pt–Ce (O@Pt–Ce).^53^ γ appearing in the temperature range of 500–600 °C is related to the desorption of oxygen from oxygen vacancies on CeO_2_ (O@CeO_2_).^51^

It has been reported that the rod-shaped ceria has both higher oxygen storage capacity and release capabilities.^54,55^ The concentration of oxygen vacancies on the surface of the catalyst has been evaluated by oxygen pulse adsorption and the OSC_surface_ values are listed in Table 2. The Pt/CeO_2_-r has a much higher OSC_surface_ value of 355.9 μmol·[O]·g^–1^ than that of Pt/CeO_2_-o of 24.3 μmol·[O]·g^–1^, which corresponds to the result of XPS and Raman. Combined with the performance of propane oxidation, it shows that the higher concentration of oxygen vacancies and mobility of oxygen species will not promote propane oxidation as reported before. On the contrary, the higher concentration of defects on the ceria might favor the formation of platinum oxide.^28,56^

The aforementioned results point out that the exposed facets of CeO_2_ have an effect on the chemical state of Pt, which in turn impacts the activity of Pt/CeO_2_ for propane oxidation. Strong Pt–Ceria interaction and high oxygen mobility will lead to a tendency of metal oxidation, thus reducing the activity of propane oxidation. In contrast, in Pt/CeO_2_-o, the weak metal–support interaction and lower concentration of oxygen vacancies promote the existence of metallic Pt (Pt^0^), which is the key reason for the improved activity in the oxidation of propane, as shown in Figure S5.

**Figure 7:**
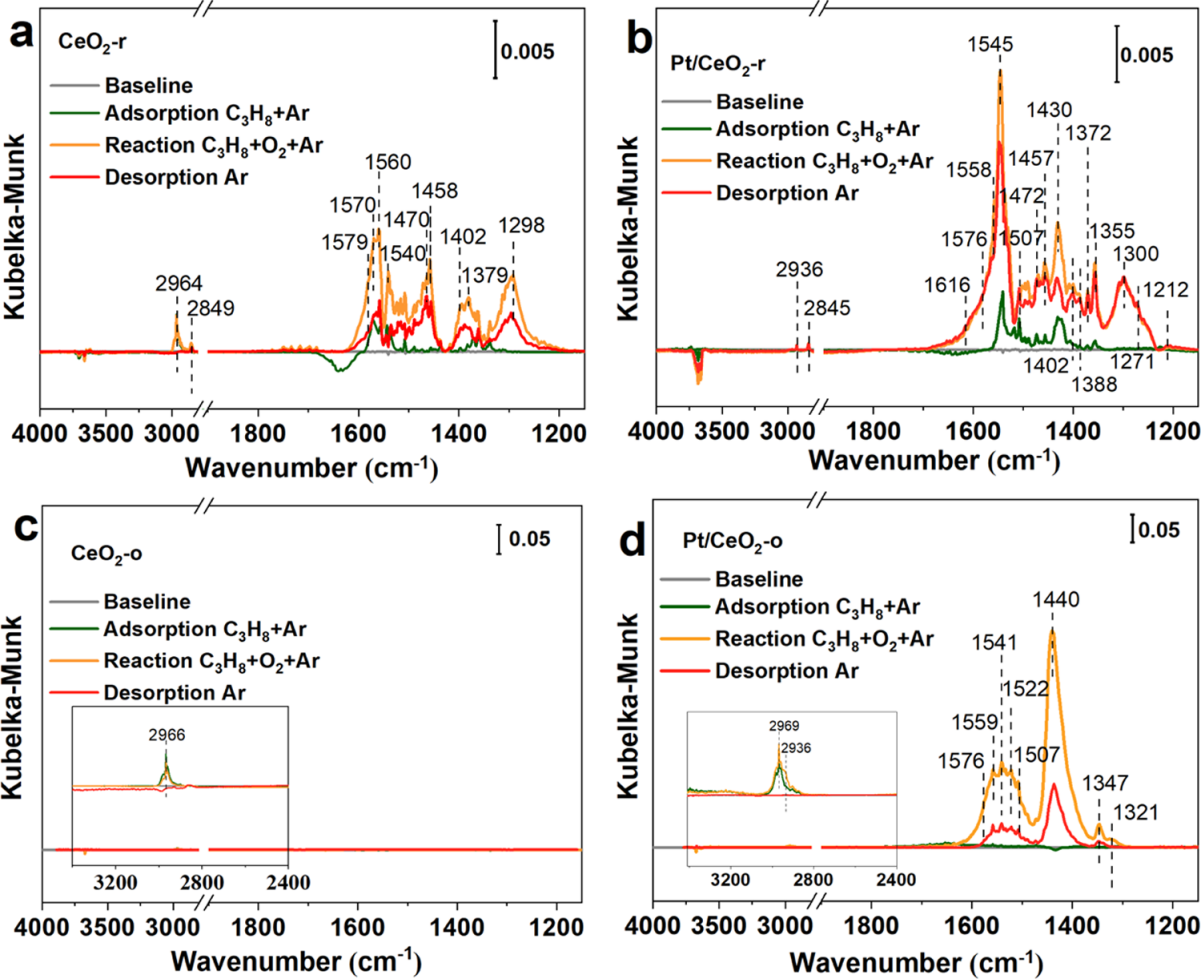
*In situ* DRIFTS of C_3_H_8_ adsorption (0.2% C_3_H_8_ in Ar, green line), C_3_H_8_ + O_2_ reaction (0.2% C_3_H_8_–2% O_2_ in Ar, orange line), desorption (Ar, red line) on CeO_2_-r (a), Pt/CeO_2_-r (b), CeO_2_-o (c), and Pt/CeO_2_-o (d) at 220 °C.

*In situ* diffuse reflectance infrared Fourier transform spectroscopy (DRIFTS) offers the possibility to reveal the influence of cerium dioxide morphology on the propane oxidation mechanism. Figure 7a–d shows the DRIFTS of the propane adsorption, oxidation, and desorption over the CeO_2_-r, CeO_2_-o, Pt/CeO_2_-r, and Pt/CeO_2_-o catalysts at 220 °C. Before the adsorption of C_3_H_8_, the catalysts were treated in Ar at 220 °C for 40 min to clean the surface of the samples.

For pure CeO_2_-r, when a feed gas of 0.2% C_3_H_8_/Ar was introduced in the chamber for 30 min, adsorption peaks of 2964 and 2849 cm^–1^ attributed to C–H vibrations of gaseous C_3_H_8_ appear.^11,57^ Furthermore, multiple adsorption bands assigned to carbonate or carboxylate species appeared between 1700 and 1200 cm^–1^, suggesting that propane can react with surface oxygen species on CeO_2_-r.^11,57^ The 1560, 1470, and 1458 cm^–1^ peaks can be attributed to ν_as_(COO), ν_s_(COO), and ν_as_(CH_3_) of propionate (CH_3_CH_2_COO^–^) species, respectively.^21^ 1579 cm^–1^ (ν_as_(COO)) and 1402 cm^–1^ (ν_s_(COO)) peaks are associated with the formation of formate (HCOO^–^). 1570 and 1460 cm^–1^ are most likely from the asymmetry and symmetry of COO^–^ in acetate (CH_3_COO^–^).^58^ The bands at 1379, 1298, and 1540 cm^–1^ can been attributed to δ_s_(CH_3_), ν(C–O), and ν_as_(COO), respectively.^15^ These results show that the lattice oxygen on the surface of CeO_2_-r can provide active oxygen species for C_3_H_8_ oxidation, resulting in the cleavage of α-H in C_3_H_8_ to generate propionate, followed by further decomposition to formate and acetate. Subsequently, replacing the feed gas with C_3_H_8_/O_2_/Ar, an obvious increase in the intensities of these intermediate species was observed, indicating that gaseous oxygen can be activated on the surface vacancies of rod-shaped CeO_2_ and react with adsorbed propane.^10^ Finally, the feed gas was changed to Ar for 30 min, and the intensity of all bands decreased by half. However, when CeO_2_-o was exposed to C_3_H_8_/Ar and C_3_H_8_/O_2_/Ar, only gaseous propane adsorption was detected, unlike CeO_2_-r, further indicating that surface oxygen on CeO_2_-o is less active compared to that on rod-shaped CeO_2_, which is consistent with the results of H_2_-TPR.

For Pt/CeO_2_-r catalysts, bands between 2850 and 3000 cm^–1^ can be attributed to gaseous propane. In addition, the assignment of the adsorption bands that appeared from 1200 to 1700 cm^–1^ is listed in Table S1. The bands at 1558 (ν_as_(COO)), 1472 (ν_s_(COO)), 1388 (ν_s_(CH_3_)), and 1457 cm^–1^ (ν_as_(CH_3_)) are consistent with propionate (CH_3_CH_2_COO^–^) species. The peaks at 1558 and 1430 cm^–1^ are most likely associated with asymmetric and symmetric stretching of bidentate acetate (CH_3_COO^–^) species.^59^ The bands at 1576/1545 cm^–1^ (ν_as_(COO)) and 2845 cm^–1^ (CH) confirm the formation of formate species (HCOO^–^).^42,57^ Similar intermediate species were observed on Pt/CeO_2_-r, but the intensities are stronger compared with CeO_2_-r, which indicates that the propane is activated at the interface of Pt–CeO_2_-r following the same reaction pathway as that on CeO_2_-r, but the more active oxygen at interface promotes the formation of intermediates. Additionally, the intensity of the adsorption peaks increased significantly when O_2_ was included in the feed gas, and bands at 1616, 1338, 1472, and 1374 cm^–1^ ascribed to C=C, CH_3_, CH_2_, and CH of propylene species (CH_3_CH=CH_2_) were detected in addition to the surface species of propionate, formate, and acetate.^60^ Meanwhile, bands at 1616, 1558, 1457, 1372, 1300, and 1212 cm^–1^ show the formation of acrylate groups (CH_2_CHCOO^–^) due to the partial oxidation of propylene species. The band at 1507 cm^–1^ is attributed to ν_as_(COO).^12^ In addition to the oxidation of propane at the interface between CeO_2_ and Pt, the large increase in the peak intensity and the formation of new intermediate species suggest the presence of O_2_ in the inlet opening a new reaction route on Pt NPs. The adsorbed propane at Pt sites was oxidized by the active oxygen species to produce formate and acetate through propylene and acrylate. The absence of a significant drop after Ar purging indicates that these intermediate species are strongly adsorbed on the surface of Pt/CeO_2_-r. Moreover, these results showed that the surface lattice oxygen of CeO_2_ is involved in the propane oxidation and reacts with activated C_3_H_8_ at the Pt–CeO_2_ interface, which agrees with the Mars–Van Krevelen (MvK) mechanism.^12^ Meanwhile, the reaction of propane oxidation also occurs on the metallic Pt surface away from the CeO_2_ carrier, where the oxygen and propane adsorb and react on the surface of metallic Pt. Therefore, for the Pt/CeO_2_-r sample, both reaction pathways proceed simultaneously during the oxidation of propane.

For the Pt/CeO_2_-o catalyst, the presence of Pt greatly facilitates the activation of propane and oxygen. When the Pt/CeO_2_-o is exposed to pure propane for 30 min, there are no carbonate species (1200–1700 cm^–1^) on the catalytic surface, indicating that the oxygen from CeO_2_ did not participate in the reaction even after loading Pt, or the adsorption intensity of the intermediate species is low, which is different from CeO_2_-r and Pt/CeO_2_-r. When the C_3_H_8_/O_2_/Ar mixed gas is introduced, obvious acetate and formate species are observed,^53,57,59,61^ which indicates that the presence of O_2_ in the inlet can promote the adsorption and activation of propane. In addition, given the fact that acrylics, acrylate, or propionate species detected on Pt/CeO_2_-r are not detected over the Pt/CeO_2_-o catalysts, which indicates that the oxidation and reaction of intermediates including C–C cleavage is significantly enhanced on Pt/CeO_2_-o. Combined with metallic Pt in XPS and H_2_-TPR, the fast oxidation and decomposition of intermediates should be relevant to the higher ratio of Pt^0^.^62^ Therefore, for the Pt/CeO_2_-o sample, the reaction pathway in which oxygen and propane are activated on the metallic Pt NPs should dominate. At the desorption stage, the intensities of all of the bands decreased sharply, indicating that those intermediate species easily desorbed from the surface of metallic Pt. This also demonstrates that for Pt/CeO_2_-r, the accumulation of intermediates should occur at the Pt–support interface. The accumulation of intermediates, formates, etc., at the interface of Pt–CeO_2_ leads to the blocking of the reaction pathway on the Pt–CeO_2_ interface, which reduces the number of available Pt active sites and thus decreases the activity of propane oxidation. The deactivation of Pt/CeO_2_-r during the long-term stability may be caused by the strong adsorption of intermediates blocking the active sites. O_2_-TPO was performed to research the carbonaceous deposits on spent Pt/CeO_2_ after a long-term stability test, as shown in Figure S6. The results show that the CO_2_ signal area detected on the used Pt/CeO_2_-r sample is 9.7 × 10^–9^, which is much higher than the area of the CO_2_ signal detected on the Pt/CeO_2_-o, which is 6.7 × 10^–10^. In order to exclude the effect of the surface area, the relative carbonate content per unit area was calculated according to the surface area of Pt/CeO_2_-r and Pt/CeO_2_-o catalysts. The relative carbonate content per unit area on Pt/CeO_2_-r was 1.37 times higher than that of the Pt/CeO_2_-o catalyst. The results for O_2_-TPO are consistent with those of DRIFTS, which proves that the strong adsorption and accumulation of intermediates at the Pt–CeO_2_ interface of Pt/CeO_2_-r sample block the active sites.

**Figure 8:**
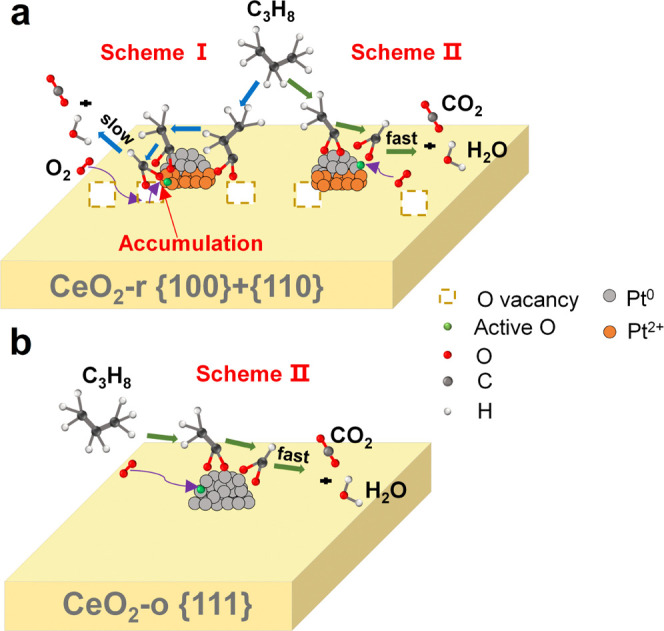
Reaction pathway scheme for C_3_H_8_ oxidation on Pt/CeO_2_-r (a) and Pt/CeO_2_-o (b).

Based on these results, it is demonstrated that the morphology of CeO_2_ affects the reaction routes of propane oxidation. A scheme of the propane oxidation on the Pt/CeO_2_ catalyst is shown in Figure 8. For Pt/CeO_2_-o, the scheme II reaction route was followed, and gas-phase oxygen and propane are adsorbed and activated on the surface of metallic Pt nanoparticles. Then, the adsorbed propane is oxidized to acetate and formate species, and further reaction produces CO_2_ and H_2_O. Meanwhile, the intensity of surface formate species is higher than acetate species on Pt/CeO_2_-r, but the results on Pt/CeO_2_-o are reversed, which indicates that metallic platinum on the {111} facet can accelerate the oxidation of formate species resulting in lower intensity of formate than acetate species. The interface between Pt and CeO_2_ originates from the highly active {110} facet of CeO_2_, making the propane reaction mechanism on Pt/CeO_2_-r more complex than that on Pt/CeO_2_-o. The reaction pathway of propane oxidation at the interface of Pt–CeO_2_ (scheme I) and on the metallic Pt NPs (scheme II, similar as Pt/CeO_2_-o) are involved simultaneously on Pt/CeO_2_-r catalyst. At the interface between Pt and CeO_2_, gaseous propane is activated and reacts with active oxygen from the Pt–CeO_2_ interface to form propylene, acrylate, and propionate species. These species then oxidized and decomposed into acetate and formate, finally producing CO_2_ and H_2_O. Due to the strong interaction between Pt and CeO_2_-r, the Pt at the interface is partially oxidized, reducing the rate of decomposition of the intermediate species, which results in the accumulation of propylene, acrylate, and propionates acetate and formate species, with formate accumulating most severely. As a result, it is considered that the accumulation of intermediate species at the interface blocked the route at the Pt–CeO_2_ interface, which led to a decrease in the atomic utilization of Pt and consequently a decrease in catalyst activity. Therefore, the superior catalytic activity of propane oxidation over Pt/CeO_2_-o compared to Pt/CeO_2_-r is realized through the higher Pt utilization. The mechanism study helps to understand the behavior of adsorption, reaction, and desorption of propane over the different nanoshaped CeO_2_ supporting Pt NPs.

## 4 Conclusions

In this work, nanorod and nano-octahedral CeO_2_ loaded with uniformly sized Pt NPs for propane oxidation were investigated. It was found that the exposed Pt/CeO_2_-o {111} facets exhibited remarkable performance for propane combustion with higher specific reaction rate and TOF values compared with Pt/CeO_2_-r, mainly exposing the {110} and {100} facets. In addition, the Pt/CeO_2_-o has excellent long-term stability for 50 h. Detailed investigations reveal that the activity of propane oxidation is related to the chemical state of Pt and metallic Pt is beneficial for propane oxidation. A higher concentration of metallic Pt species was observed on the {111} facets of CeO_2_ because of weak interaction between Pt and CeO_2_ and low concentration of defects. Propionate, propylene, and acrylate species containing three-carbon intermediates were detected on Pt/CeO_2_-r, but not on Pt/CeO_2_-o, confirming that metallic Pt plays an important role in the cleavage of C–C bonds of adsorbed propane. The major intermediate species on Pt/CeO_2_-r is formate; however, acetate becomes the primary intermediate on the Pt/CeO_2_-o surface due to the rapid decomposition of formate. Besides, a dominant scheme II reaction pathway is observed on Pt/CeO_2_-o, where oxygen and propane are activated on the metallic Pt NPs. Although two reaction routes at the Pt–CeO_2_ interface (scheme I) and metallic Pt (scheme II) coexist over the Pt/CeO_2_-r catalyst, the accumulation of intermediate species at the interface hinders the reaction pathway of propane oxidation at the Pt–CeO_2_ interface, leading to a decrease in Pt utilization and thus reducing the activity of the Pt/CeO_2_-r catalyst.

## Supporting Information Available

Preparation of Pt nanoparticles, and Pt/CeO_2_ catalysts; TEM image of as-synthesized Pt nanoparticles and size distribution; catalytic activity of C_3_H_8_ oxidation on Pt/CeO_2_-r, Pt/CeO_2_-o, and Pt/CeO_2_-r-IWI samples with and without 3% H_2_O; O_2_-TPO of Pt/CeO_2_ samples; Raman spectra of CeO_2_ and Pt/CeO_2_; and relationship between TOF and the concentration of Pt^0^, the concentration of Ce^3+^, the intensity ratio of formate/acetate species and the table of frequencies of functional groups present on different catalysts analyzed by *in situ* DRIFTS (PDF)
