# Supplementary material for: Preformed Pt Nanoparticles Supported on Nanoshaped CeO2 for Total Propane Oxidation
Source: ACS Appl Nano Mater. 2023 Aug 15;6(16):15073–84. doi: 10.1021/acsanm.3c02688 (PMC10464920; doi:10.1021/acsanm.3c02688)
Supplement: Supplementary file 1 — an3c02688_si_001.pdf [file an3c02688_si_001.pdf]

Supporting information

Preformed Pt Nanoparticles Supported on Nanoshaped CeO_2 for Total Propane Oxidation

Shasha Ge[1,2], Yufen Chen[2], Xuan Tang[1], Yali Shen[1], Yang Lou[3], Li Wang[1], Yun Guo[1,*], Jordi Llorca[2,*]

[1] Key Laboratory for Advanced and Research Institute of Industrial catalysis, School of Chemistry & Molecular Engineering, East China University of Science and Technology, Shanghai 200237, P. R. China.

[2] Institute of Energy Technologies, Department of Chemical Engineering and Barcelona Research Center in Multiscale Science and Engineering, Universitat Politècnica de Catalunya, EEBE, Eduard Maristany 10-14, 08019 Barcelona, Spain.

[3] Key Laboratory of Synthetic and Biological Colloids, Ministry of Education, School of Chemical and Material Engineering, Jiangnan University, Wuxi, Jiangsu 214122, P. R. China

[*] Corresponding authors. E-mail addresses: yunguo@ecust.edu.cn; jordi.llorca@upc.edu

1. Catalyst Preparation

1.1. The preparation of Pt nanoparticles

The preparation of Pt nanoparticles was described in detail in Ref.[2] Firstly, 60 mg of chloroplatinic acid (H_2PtCl_6) and 220 mg of NaOH were dissolved in 20 mL of ethylene glycol under magnetic stirring, and then 10 mL of deionized water was added. The solution was heated in an oil bath at 90 °C for 2h under the protection of nitrogen gas, and when the colour of the solution changed from light yellow to brown, it indicated the formation of Pt nanoparticles. After cooling, 0.3 $mol \cdot L^{-1}$ of HCl was added to extract the Pt nanoparticles. Finally, the nanoparticles were dispersed in ethanol for the next loading step.

1.2. The preparation of Pt/CeO_2 catalysts

The Pt/CeO_2 catalysts were prepared by the adsorption method. Typically, CeO_2 powder was ultrasonically dispersed in 30 mL of ethanol solution, and then a certain amount of Pt nanoparticles prepared above was added and stirred overnight at room temperature. After centrifugal separation, the solid powder was washed with ethanol and deionized water. A saturated silver nitrate solution was used to detect the removal of Cl^-. Finally, the solid was dried in a vacuum oven at 60 °C for 8 h and calcined at 400 °C for 2 h in N_2. The catalysts were labelled as Pt/CeO_2-r and Pt/CeO_2-o. The content of Pt was determined by ICP-AES.

1.3. The preparation of Pt/CeO_2-r-IWI catalyst

The Pt/CeO_2-r-IWI sample was prepared by incipient wetness impregnation. The CeO_2-r carrier was placed in a certain amount of aqueous platinum (II) nitrate solution. Then, the

mixture was dried overnight in the oven at 80 °C. Finally, the dried powder was calcinated in air at 400 °C for 4 h to get Pt/CeO_2-r-IWI catalyst.

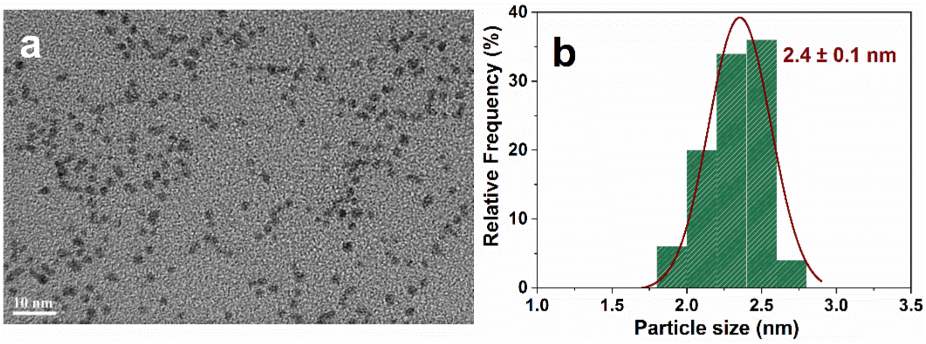

Figure S1. TEM image of as-synthesized Pt nanoparticles (a) and size distribution of Pt nanoparticles (b).

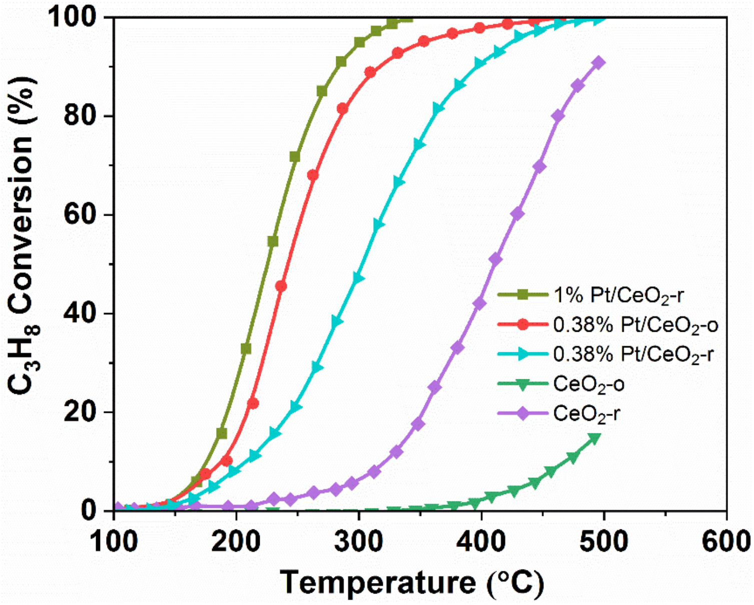

Figure S2. Catalytic activity of C_3H_8 oxidation on various samples.

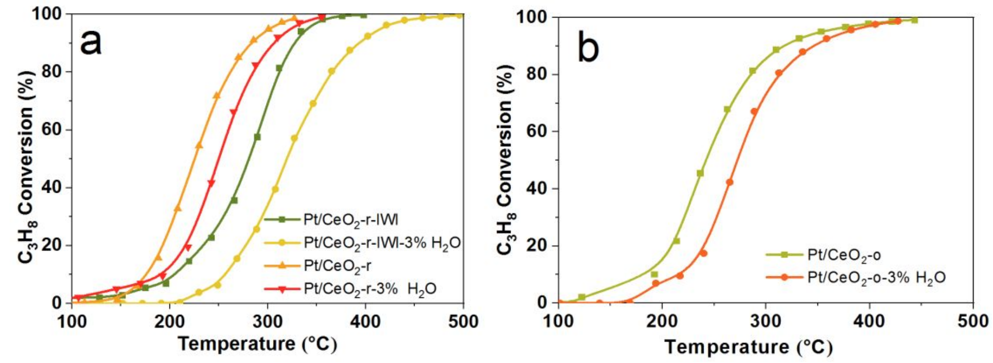

Figure S3. Catalytic activity of Pt/CeO_2-r, Pt/CeO_2-r-IWI (a) and Pt/CeO_2-o (b) with and without 3% H_2O.

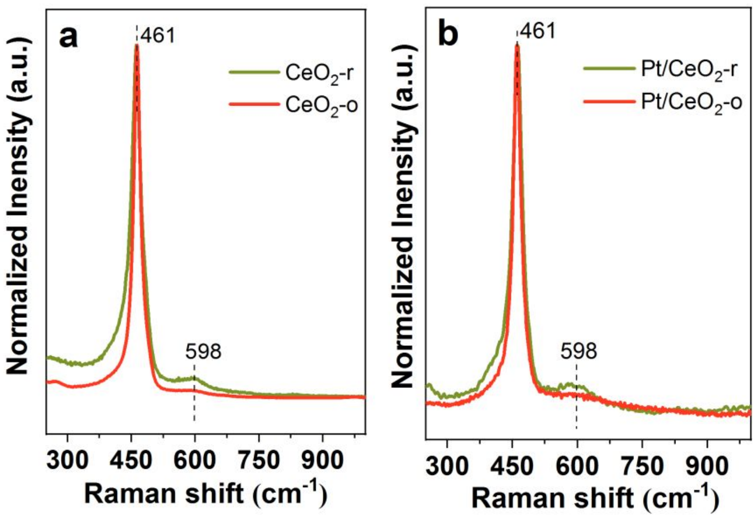

Figure S4. The Raman spectra of CeO_2 (a) and Pt/CeO_2 (b).

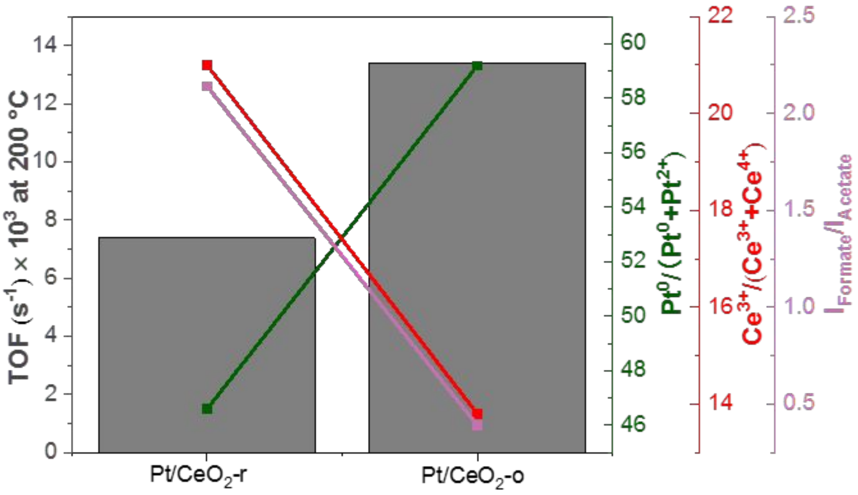

Figure S5. The relationship between TOF (at 200 °C) and the concentration of Pt^0, the concentration of Ce^{3+} and the intensity ratio of formate/acetate species.

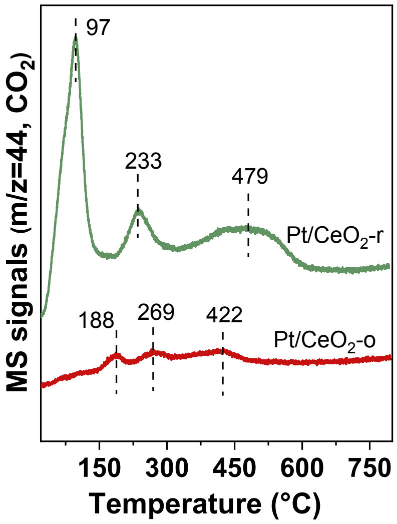

Figure S6. TPO of Pt/CeO_2-r and Pt/CeO_2-o samples after long-term tests.

Table S1. Frequencies of functional groups present on different catalysts analyzed by *in-situ* DRIFTS.

Band/cm^{-1}	Assignment	Species	References
2900-2950	$\nu_{as}(CH_3)$	$C_3H_8(g)$	[1,3–5]
2936	$\nu_{as}(CH_2)$	--	[6–9]
2849	$\nu_{as}(CH)$	--	[6–8]
1616	$\nu(C=C)$	$CH_3CH=CH_2$	[10–13]
1388	$\nu_s(CH_3)$		
1457	$\nu_{as}(CH_3)$		
1372	$\nu_s(CH)$		
1558	$\nu_{as}(COO)$	$CH_3CH_2COO^-$	[14–16]
1472	$\nu_s(COO)$		
1388	$\nu_s(CH_3)$		
1457	$\nu_{as}(CH_3)$		
1616	$\nu(C=C)$	CH_2CHCOO^-	[11–13,17–19]
1558	$\nu_{as}(COO)$		
1472	$\nu_s(COO)$		
1212/1271	$\delta_{as}(CH=)$		
1300	$\nu(C\text{-}O)$		
2845	$\nu(C\text{-}H)$	$HCOO^-$	[5,15,17,20–24]
1576/1545	$\nu_{as}(COO)$		
1402	$\nu_s(COO)$		
1430/1440	$\nu_s(COO)$	CH_3COO^-	[15,17,20,21,24]
1559	$\nu_{as}(COO)$		
1347	$\delta_{as}(CH_3)$		

REFERENCES

(1) Hu, Z.; Liu, X.; Meng, D.; Guo, Y.; Guo, Y.; Lu, G. Effect of Ceria Crystal Plane on the Physicochemical and Catalytic Properties of Pd/Ceria for CO and Propane Oxidation. *ACS Catal.* **2016**, *6* (4), 2265–2279.

(2) Peng, R.; Li, S.; Sun, X.; Ren, Q.; Chen, L.; Fu, M.; Wu, J.; Ye, D. Size Effect of Pt Nanoparticles on the Catalytic Oxidation of Toluene over Pt/CeO_2 Catalysts. *Appl. Catal. B Environ.* **2018**, *220*, 462–470.

(3) Hu, Z.; Wang, Z.; Guo, Y.; Wang, L.; Guo, Y.; Zhang, J.; Zhan, W. Total Oxidation of Propane over a Ru/CeO_2 Catalyst at Low Temperature. *Environ. Sci. Technol.* **2018**, *52* (16), 9531–9541.

(4) Liao, W. M.; Fang, X. X.; Cen, B. H.; Chen, J.; Liu, Y. R.; Luo, M. F.; Lu, J. Q. Deep Oxidation of Propane over WO_3 - Promoted Pt/BN Catalysts: The Critical Role of Pt - WO_3 Interface. *Appl. Catal. B Environ.* **2020**, *272*, 118858.

(5) Wu, S.; Liu, H.; Huang, Z.; Xu, H.; Shen, W. $Mn_1Zr_xO_y$ Mixed Oxides with Abundant Oxygen Vacancies for Propane Catalytic Oxidation: Insights into the Contribution of Zr Doping. *Chem. Eng. J.* **2023**, *452*, 139341.

(6) Huang, Z.; Cao, S.; Yu, J.; Tang, X.; Guo, Y.; Guo, Y.; Wang, L.; Dai, S.; Zhan, W. Total Oxidation of Light Alkane over Phosphate-Modified Pt/CeO_2 Catalysts. *Environ. Sci. Technol.* **2022**, *56* (13), 9661–9671.

(7) Hu, Z.; Qiu, S.; You, Y.; Guo, Y.; Guo, Y.; Wang, L.; Zhan, W.; Lu, G. Hydrothermal Synthesis of $NiCeO_x$ Nanosheets and Its Application to the Total Oxidation of Propane. *Appl. Catal. B Environ.* **2018**, *225*, 110–120.

(8) Held, A.; Kowalska-Kuś, J.; Nowińska, K. Propane-to-Propene Oxide Oxidation on Silica-Supported Vanadium Catalysts with N_2O as an Oxidant. *J. Catal.* **2016**,

336, 23–32.

(9) Huang, Z.; Ding, J.; Yang, X.; Liu, H.; Song, P.; Guo, Y.; Guo, Y.; Wang, L.; Zhan, W. Highly Efficient Oxidation of Propane at Low Temperature over a Pt-Based Catalyst by Optimization Support. *Environ. Sci. Technol.* **2022**, *56* (23), 17278–17287.

(10) Wang, C.; Feng, F.; Du, J.; Zheng, T.; Pan, Z.; Zhao, Y. Activation of Surface Lattice Oxygen in Ceria Supported Pt/Al_2O_3 Catalyst for Low-Temperature Propane Oxidation. *ChemCatChem*. **2019**, 2054–2057.

(11) Gerei, S. V.; Rozhkova, E. V.; Gorokhovatsky, Y. B. Propylene and Oxygen Chemisorption on Cupric Oxide and Cuprous Oxide Catalysts. *J. Catal.* **1973**, *28* (3), 341–350.

(12) Koziej, D.; Bârsan, N.; Hoffmann, V.; Szuber, J.; Weimar, U. Complementary Phenomenological and Spectroscopic Studies of Propane Sensing with Tin Dioxide Based Sensors. *Sensors Actuators, B Chem.* **2005**, *108*, 75–83.

(13) Wu, X.; Zhang, L.; Weng, D.; Liu, S.; Si, Z.; Fan, J. Total Oxidation of Propane on Pt/WO_x/Al_2O_3 Catalysts by Formation of Metastable $Pt^{\delta+}$ Species Interacted with WO_x Clusters. *J. Hazard. Mater.* **2012**, *225–226*, 146–154.

(14) Shan, S.; Li, J.; Maswadeh, Y.; O'Brien, C.; Kareem, H.; Tran, D. T.; Lee, I. C.; Wu, Z. P.; Wang, S.; Yan, S.; Cronk, H.; Mott, D.; Yang, L.; Luo, J.; Petkov, V.; Zhong, C. J. Surface Oxygenation of Multicomponent Nanoparticles toward Active and Stable Oxidation Catalysts. *Nat. Commun.* **2020**, *11* (1), 1–9.

(15) O'Brien, C. P.; Lee, I. C. A Detailed Spectroscopic Analysis of the Growth of Oxy-Carbon Species on the Surface of Pt/Al_2O_3 during Propane Oxidation. *J. Catal.* **2017**, *347*, 1–8.

(16) Finocchio, E.; Busca, G.; Lorenzelli, V.; Escribano, V. S. FTIR Studies on the

Selective Oxidation and Combustion of Light Hydrocarbons at Metal Oxide Surfaces. Part 2. - Propane and Propene Oxidation on Co_3O_4. *J. Chem. Soc. - Faraday Trans.* **1996**, *92* (9), 1587–1593.

(17) Liu, Y.; Cai, Y.; Tang, X.; Shao, C.; You, Y.; Wang, L.; Zhan, W.; Guo, Y.; Zhao, Y. K.; Guo, Y. Insight into the Roles of Pd State and CeO_2 Property in C_3H_8 Catalytic Oxidation on Pd/CeO_2. *Appl. Surf. Sci.* **2022**, *605*, 154675.

(18) Concepción, P.; Botella, P.; Nieto, J. M. L. Catalytic and FT-IR Study on the Reaction Pathway for Oxidation of Propane and Propylene on V- Or Mo-V-Based Catalysts. *Appl. Catal. A Gen.* **2004**, *278* (1), 45–56.

(19) Wang, Z.; Huang, Z.; Brosnahan, J. T.; Zhang, S.; Guo, Y.; Guo, Y.; Wang, L.; Wang, Y.; Zhan, W. Ru/CeO_2 Catalyst with Optimized CeO_2 Support Morphology and Surface Facets for Propane Combustion. *Environ. Sci. Technol.* **2019**, *53* (9), 5349–5358.

(20) Zhang, T.; Lang, X.; Dong, A.; Wan, X.; Gao, S.; Wang, L.; Wang, L.; Wang, W. Difference of Oxidation Mechanism between Light C3-C4 Alkane and Alkene over Mullite YMn_2O_5 Oxides' Catalyst. *ACS Catal.* **2020**, *10* (13), 7269–7282.

(21) Li, G.; Li, N.; Sun, Y.; Qu, Y.; Jiang, Z.; Zhao, Z.; Zhang, Z.; Cheng, J.; Hao, Z. Efficient Defect Engineering in Co-Mn Binary Oxides for Low-Temperature Propane Oxidation. *Appl. Catal. B Environ.* **2021**, *282*, 119512.

(22) Pozdnyakova, O.; Teschner, D.; Wootsch, A.; Kröhnert, J.; Steinhauer, B.; Sauer, H.; Toth, L.; Jentoft, F. C.; Knop-Gericke, A.; Paál, Z.; Schlögl, R. Preferential CO Oxidation in Hydrogen (PROX) on Ceria-Supported Catalysts, Part I: Oxidation State and Surface Species on Pt/CeO_2 under Reaction Conditions. *J. Catal.* **2006**, *237* (1), 1–16.

(23) Durand, J. P.; Senanayake, S. D.; Suib, S. L.; Mullins, D. R. Reaction of Formic

Acid over Amorphous Manganese Oxide Catalytic Systems: An in Situ Study. *J. Phys. Chem. C* **2010**, *114* (47), 20000–20006.

(24) Wu, J.; Chen, B.; Yan, J.; Zheng, X.; Wang, X.; Deng, W.; Dai, Q. Ultra-Active Ru Supported on CeO_2 Nanosheets for Catalytic Combustion of Propane: Experimental Insights into Interfacial Active Sites. *Chem. Eng. J.* **2022**, *438*, 135501.
